# A Smart Ski Pole for Skiing Pattern Recognition and Quantification Application

**DOI:** 10.3390/s24165291

**Published:** 2024-08-15

**Authors:** Yangyanhao Guo, Renjie Ju, Kunru Li, Zhiqiang Lan, Lixin Niu, Xiaojuan Hou, Shuo Qian, Wei Chen, Xinyu Liu, Gang Li, Jian He, Xiujian Chou

**Affiliations:** 1 Science and Technology on Electronic Test and Measurement Laboratory, School of Instrument and Electronics, North University of China, Taiyuan 030051, China; s202206044@st.nuc.edu.cn (Y.G.); sz202206019@st.nuc.edu.cn (R.J.); s202306056@st.nuc.edu.cn (K.L.); sz202106125@st.nuc.edu.cn (L.N.); houxiaojuan@nuc.edu.cn (X.H.); sz202106192@st.nuc.edu.cn (W.C.); sz202106136@st.nuc.edu.cn (X.L.); chouxiujian@nuc.edu.cn (X.C.); 2 School of Future Science and Engineering, Soochow University, Suzhou 215299, China; 3 School of Software, North University of China, Taiyuan 030051, China; qianshuo@nuc.edu.cn; 4 School of Physical Education, Tianjin University of Sport, Tianjin 301600, China; lig1117@126.com

**Keywords:** smart ski poles, cross-country skiing, inertial measurements, skiing patterns recognition

## Abstract

In cross-country skiing, ski poles play a crucial role in technique, propulsion, and overall performance. The kinematic parameters of ski poles can provide valuable information about the skier’s technique, which is of great significance for coaches and athletes seeking to improve their skiing performance. In this work, a new smart ski pole is proposed, which combines the uniaxial load cell and the inertial measurement unit (IMU), aiming to provide comprehensive data measurement functions more easily and to play an auxiliary role in training. The ski pole can collect data directly related to skiing technical actions, such as the skier’s pole force, pole angle, inertia data, etc., and the system’s design, based on wireless transmission, makes the system more convenient to provide comprehensive data acquisition functions, in order to achieve a more simple and efficient use experience. In this experiment, the characteristic data obtained from the ski poles during the Double Poling of three skiers were extracted and the sample *t*-test was conducted. The results showed that the three skiers had significant differences in pole force, pole angle, and pole time. Spearman correlation analysis was used to analyze the sports data of the people with good performance, and the results showed that the pole force and speed (*r* = 0.71) and pole support angle (*r* = 0.76) were significantly correlated. In addition, this study adopted the commonly used inertial sensor data for action recognition, combined with the load cell data as the input of the ski technical action recognition algorithm, and the recognition accuracy of five kinds of cross-country skiing technical actions (Diagonal Stride (DS), Double Poling (DP), Kick Double Poling (KDP), Two-stroke Glide (G2) and Five-stroke Glide (G5)) reached 99.5%, and the accuracy was significantly improved compared with similar recognition systems. Therefore, the equipment is expected to be a valuable training tool for coaches and athletes, helping them to better understand and improve their ski maneuver technique.

## 1. Introduction

Using smart sports equipment has grown to be a significant trend in supplemental training for sports in recent years [1]. A variety of smart sports equipment has been developed as a result of technological advancements in order to improve training, performance, and overall athlete development [2,3,4]. These sophisticated gadgets and tools may capture a variety of athletic data, including speed, acceleration, and other factors, as well as track bodily functions like heart rate, respiration, and body temperature [5,6,7,8,9,10,11,12]. This data-driven training technique can be very advantageous for participants because it allows them to analyze and learn from the technical strategies of top performers, which helps them enhance their own performance [13,14].

Skiing has a long history and is now a well-liked sport all around the world. In an effort to enhance performance, research has increasingly concentrated on the numerous elements that affect the maximum exertion during skiing [15]. Skiers and researchers are both worried about a number of important variables, such as the ski pole’s inclination, the amount of the axial load, the angle at which force is applied, and others [16,17,18]. Numerous tools and techniques are used for this, such as the multi-inertial sensing-based ski attitude identification system [19,20] and the auxiliary training system [21,22,23,24,25], which uses video and inertial measurement. The widespread usage of these gadgets by the general public and enthusiasts still faces certain difficulties, though. First off, some smart sports equipment necessitates wearing devices or fastening them to equipment [26,27], which can complicate and impede training. In some cases, the time needed for setup, calibration, and ensuring correct usage [28,29] may discourage people. Second, participants’ freedom and flexibility are sometimes restricted by the bulky nature of high-precision instruments. The equipment’s size and weight limit natural motions and detract from the training process as a whole. Additionally, the equipment used for professional training frequently uses cutting-edge sensors and technology, which increases its relative cost [30,31]. To address these issues, it is crucial to create a simple, effective, and smart ski pole.

This research proposes a wireless smart ski pole; it is a valuable training tool for skiers, improving technique and maximizing performance. The pole’s sensors, equipped with an inertial measurement unit (IMU) and a uniaxial load unit, collect various data on the skier’s performance and technique. These data are then used for sample testing and correlation analysis to explore the relationship between the skier’s movement characteristics and skiing speed. The data are also fed into the SVM-based action recognition model, which is trained to distinguish five types of cross-country skiing techniques.

## 2. Methods

### 2.1. Measurement

**Figure 1:**
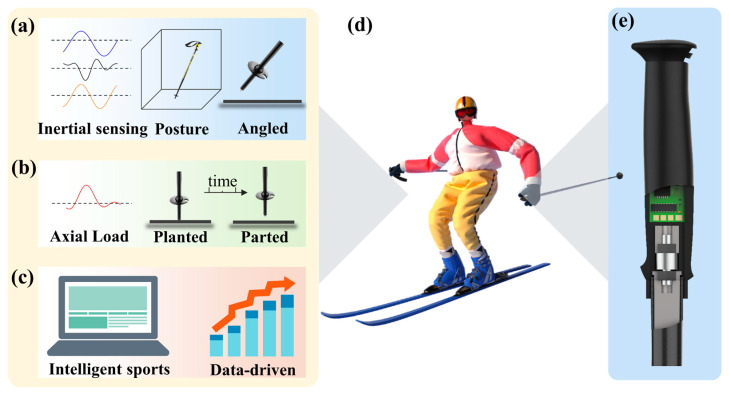
Smart ski pole functional requirements and pole design. (**a**) Inertial data measurement and ski pole model driving. (**b**) Axial load monitoring of the pole. (**c**) Intelligent sports data-driven learning. (**d**) Skier and (**e**) ski pole grip structure.

In cross-country skiing (Figure 1d), ski poles play a key role in completing relevant technical movements, so it is of great significance to study ski poles. We can collect gyroscope (Gyro), acceleration, and angle data using the inertial sensing IMU, which has the advantages of small size and high measurement precision. As seen in Figure 1a, these data can be utilized to drive a three-dimensional model to track details like the slope angle and ski pole rotation speed. It can also be visually displayed as a waveform. Additionally, because the magnitude and timing of the force impacts the skiing action, skiers are highly interested in monitoring the axial load force acting on the ski pole in the vertical direction. Skiers can better comprehend their movements, enhance their skiing technique, and maximize their performance by measuring this force and how it changes (Figure 1b). In this study, a special pole grip is created, as shown in Figure 1e, to collect data such as pole force, pole angle, and inertia data applied by the skier while skiing. This information is fed back to the skier, which can help them improve their technique and, in turn, their skiing (Figure 1c).

Ski poles must be made with durability and functionality in mind. Axial loads and inertial measurements deserve special attention in this regard. Axial load assessment is essential for skiing because it is one of the key forces that propels the skier. Key elements impacting speed are the amount of axial load, the time applied, and the timing of skiing. A micro sensor made by Hengyuan Company (Shandong, China) has a uniaxial load cell which is small in size and high in hardness, weighing only 100 g, with a rated load up to 1000 N and sensitivity up to 1.0 + 20% mV/V. It uses a bolt structure and stainless-steel material to ensure its stability and durability. The angle change in the ski pole, gyroscope data, and acceleration data, among other things, are examples of inertial measurement, which is another important factor to take into account. Each of these elements affect speed. The ICM20602 inertial sensing unit (InvenSense, San Jose, CA, USA) has a variety of range and angular output accuracy to meet the requirements of use. With the help of model-driven and data analysis, these high-precision data can be used to provide precise information to advance skiing tactics.

A 12 mm × 79 mm PCB contains the peripheral circuit, micro controller unit, power supply, and wireless transceiver module. Using the construction of the bolt axial pressure sensors and the connecting rod body, the PCB is firmly fixed inside the rod body. The grip and rod body must have a 2 mm compression space between them due to the pressure sensor’s properties. The inner wall of the grip fits exactly into the connector, improving not only the stability and dependability of the entire rod body but also reducing the chance of unneeded movement or vibration affecting the data’s correctness. The design of the grip decreases the effect of the weight of the full bar on the sense of balance once it is installed because practically all of the extra weight is focused inside the hand’s grasp.

**Figure 2:**
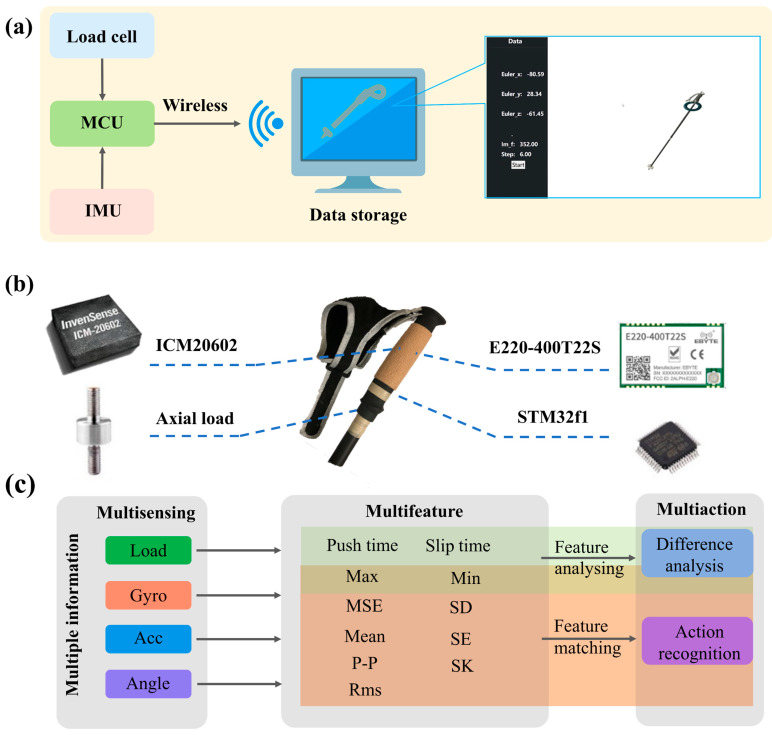
The system architecture. (**a**) Data acquisition, transmission, and storage; (**b**) the main devices selection; (**c**) the extraction of characteristic value of mean squared error (MSE), peak-to-peak (P-P), root mean square (Rms), standard deviation (SD), standard error (SE), skewness (SK), etc.

The system’s data transmission and storage are described in Figure 2a. The system can also calibrate the ski pole prior to testing in order to collect more precise data for the experiment. The collected data are transmitted wirelessly to the top computer through the Long-Range Radio (LORA) module for further analysis. A stable and efficient transmission effect can be obtained by selecting an appropriate transmission rate, and the loss of data in this case has little effect on the experimental results. Through the top computer interface of the computer, it is easier and more logical to verify the accuracy of the data. The computer can also show the size of its value. The relationship between the rod’s attitude in reality and the image is compared to determine whether the inertial IMU is calibrated, and the operations of horizontal placement, vertical placement, or swing are used to confirm the precision and timeliness of the measurement. The differentiated training analysis and classification recognition model of skiing patterns established for skiing data are shown in Figure 2c. The retrieved characteristic values come from the data collected by the fused multi-sensors. By retrieving characteristic values from different data such as axial load, rotation angle, acceleration (Acc) and angular velocity obtained by different sensors, the difference in the skiing state of athletes can be analyzed. This classification is based on several characteristic values, and different technical actions are identified.

### 2.2. Difference Comparison

The experimental subjects were three adult men who had successfully completed a cross-country skiing training program and demonstrated reliable skiing ability. All were approximately 180 cm tall, weighed 80 kg, and were 25 years old. Table 1 displays the demographic data including their performance. Skiers glided over a flat 1000-m stretch of dry field to simulate a flat section in cross-country skiing. They utilized the Double Poling (DP) technique for skiing, and the data collected from the three individuals were analyzed. The sampling rate of the ski poles is 60 Hz. Simultaneously, speed played a crucial role, and Global Positioning System (GPS) technology was used to track the skiers and ensure accurate speed measurements, providing them with real-time feedback via wearable GPS devices.

This comparison aimed primarily to discern differences in skiing actions among the three individuals using the ski poles. It sought to investigate whether significant differences existed among different individuals under the same skiing actions. Additionally, speed data collected via GPS were utilized to analyze the correlation between speed and factors such as ski pole angle and thrust strength. Through these analyses, deeper insights into performance disparities among different individuals under identical actions were gained, providing valuable references for personalized skiing training.

Since the DP technique exhibits high coordination symmetry, data from the right ski poles of the three individuals were primarily analyzed when stable. The main focus was on the magnitude of force applied during pole pushing and the planting angle. This not only provided visual observations of the skiers’ actions during pole thrusting but also facilitated the calculation of the duration of pole thrusting and skiing recovery time. To determine the pole thrusting cycle, the critical point of sudden pressure increase, i.e., the moment when the ski pole just hits the ground, was identified as the starting point of the cycle, and the starting point of the adjacent cycle was defined as the endpoint of the current cycle. Using this method, a clear definition of the pole thrusting cycle in a skiing action was established.

**Figure 3:**
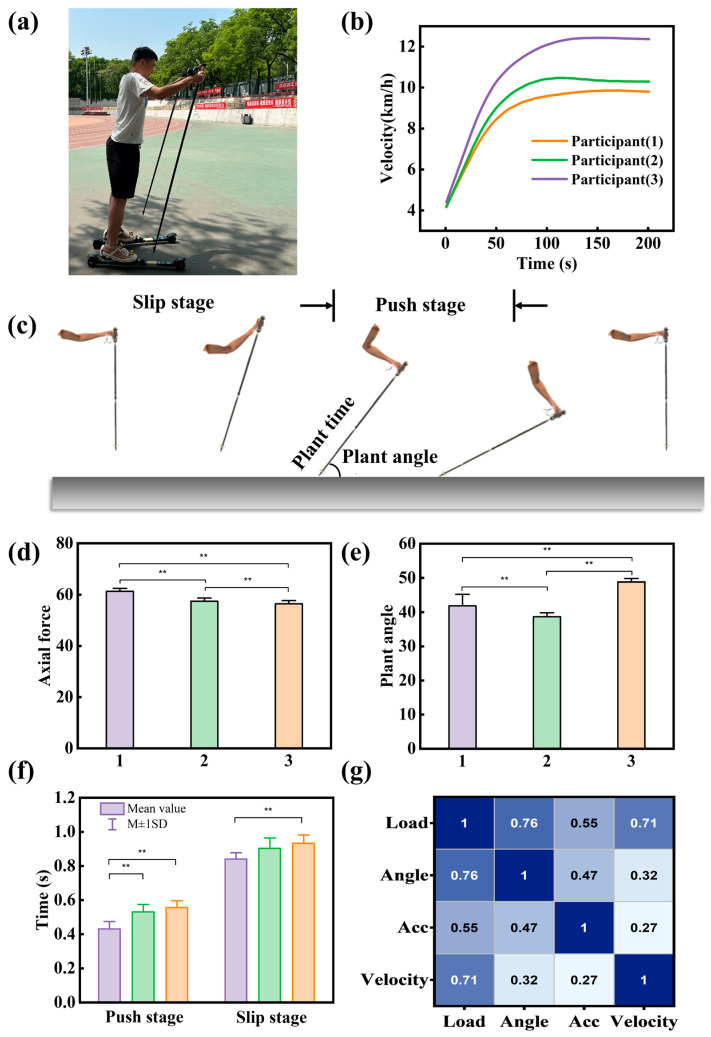
Test pole performance, correlation difference analysis. (**a**) Roller ski and site indication. (**b**) Motion speed cure of three participants. (**c**) DP technology arm motion decomposition. (**d**–**f**) Analysis of the difference in axial force, plant angle, pushing time, and slipping time, where ‘**’ means *p* ≤ 0.01. (**g**) Thermal map analysis of skiing data correlation.

Based on the defined skiing action cycles, we extracted the maximum values of pole axial force, the angle of the pole at ground contact, pole thrusting time, and skiing time from multiple skiing cycles of the three skiers. The insertion angle of the ski pole at ground contact, as depicted in Figure 3c, varied among individuals due to differences in their technical movements’ extent of extension. Therefore, when the pressure reaches its maximum value during the strut process, we record the pressure, angle, strut time, glide distance, and other relevant data. Discrepancies in the length of the poling cycle and the magnitude of poling force indicated variations in skiers’ poling strategies. Some skiers might prefer to thrust poles frequently and rapidly, while others might opt to exert greater force over a longer duration. This variability could be related to individual physical fitness, technical proficiency, and differing needs for speed control.

The formula for calculating the *t*-test statistic is as follows:

(1) $$t=\left({\bar{x}}_{1}-{\bar{x}}_{2}\right)/s_{p}\left(\sqrt{1/n_{1}+1/n_{2}}\right)$$

where ${\bar{x}}_{1}$ and ${\bar{x}}_{2}$ are the sample means of sample 1 and sample 2, respectively, *n*_1_ and *n*_2_ are the sample sizes of sample 1 and sample 2, *S_p_* is the pooled standard deviation, and *S*_1_^2^ and *S*_2_^2^ are the sample variances of sample 1 and sample 2, computed using the following formulas:

(2) $$s_{p}=\sqrt{\left(n_{1}-1\right)s_{1}^{2}+\left(n_{2}-1\right)s_{2}^{2}/\left(n_{1}+n_{2}-2\right)}$$

Then, based on the degrees of freedom (*n*_1_ + *n*_2_ − 2) and the *t*-test statistic, the corresponding *p*-value is calculated.

Next, conclusions are validated through correlation analysis. Common correlation analyses include linear correlation analysis (Pearson correlation analysis), Kendall rank correlation analysis, and rank correlation analysis (Spearman correlation analysis). Linear correlation analysis is a parametric test based on means, requiring both variables to be normally distributed quantitative variables. Kendall rank correlation analysis is specifically used for analyzing the correlation between rank variables and produces similar results to rank correlation analysis. Rank correlation analysis is a non-parametric test based on ranks, requiring both variables to be quantitative or rank variables. The normality test results indicate that the data for pressure changes, angle changes, etc., are not from normally distributed populations. Therefore, Spearman correlation analysis is used for testing. The formula for Spearman correlation analysis is as follows:

(3) $$\theta =\frac{\sum \left(R_{i}-\bar{R}\right)\left(S_{i}-\bar{S}\right)}{\sqrt{\sum {\left(R_{i}-\bar{R}\right)}^{2}{\left(S_{i}-\bar{S}\right)}^{2}}}$$

The correlation coefficient |*r*| between 0.8 and 1 indicates a high correlation, while |*r*| between 0.3 and 0.8 indicates a moderate correlation.

### 2.3. Action Recognition

**Figure 4:**
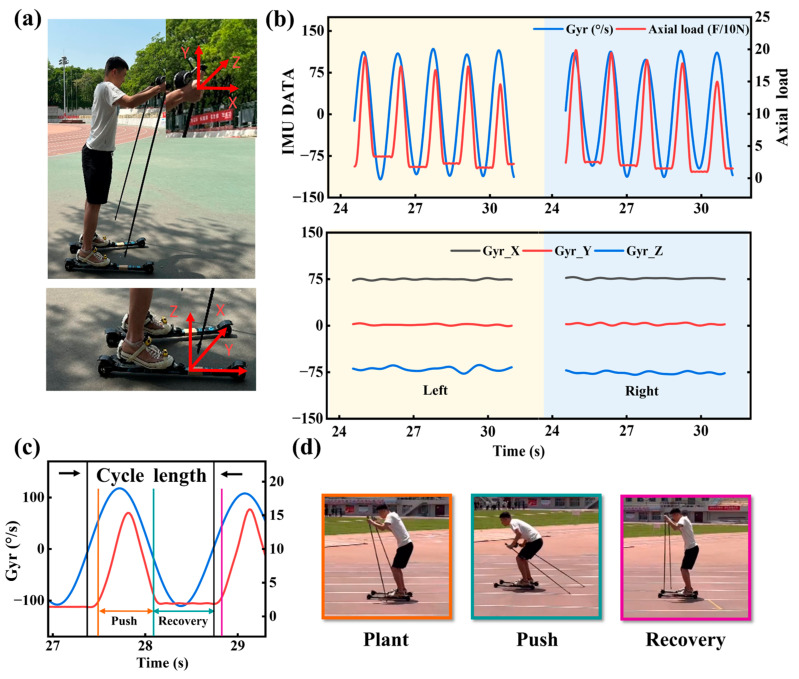
Inertial sensor axis and action interval definition. (**a**) Ski pole and roller snowboard’s inertial sensing axis. (**b**) The output data of the poles and the roller skis. (**c**) The ski period is defined based on Z-axis gyro data. (**d**) The push stage and slide stage are defined according to whether the rod is in contact with the ground.

In order to ensure the consistency of the collected data, the orientation of the ski poles and roller skis within the IMU coordinate system must be specified. First of all, both ski poles have identical sensors on the left and right, making them practically equivalent. It is crucial to remember that the IMU’s axis is set in relation to the ski poles to guarantee the reliability and consistency of the data. Figure 4a illustrates how the positive x-axis of the ski pole IMU coordinate system points to the front of the body, the positive y-axis ascends along the snow pole, and the positive z-axis of the right-hand ski pole points to the interior of the body. On the front portion of each of the two roller skis, in the free locations, were the final two IMUs.

To better prepare for the action recognition part, we extract the eigenvalues of other parts of the data on the basis of the action interval division. Based on the division of cycles of the z-axis gyroscope data from the right ski pole, feature extraction is conducted on sensor node data within intervals. This includes data from a pair of ski boards and a pair of ski poles, totaling four sensor nodes. Feature extraction follows the criteria outlined in Table 2, encompassing twelve time-domain features and three frequency-domain features, totaling 100 feature vectors. The extracted eigenvalues are used to train the classifier to realize ski pattern recognition.

When dealing with such high-dimensional nonlinear feature vectors, a support vector machine (SVM) is able to implicitly represent the data in the high-dimensional space by using kernel functions, thus avoiding the inner product of the high-dimensional feature space [32,33]. The core of SVM classification is to find a decision boundary that can separate different categories of data and has the maximum interval, and the decision boundary is usually expressed as

(4) ωx−b=0

where *x* is the input feature vector, *w* is the weight vector, and *b* is the bias term, where the values of *w* and *b* can be obtained by solving the quadratic optimization problem:

(5) $$\begin{array}{c} \underset{w,\xi ,b}{min}\frac{1}{2}∥w∥+\frac{1}{cn}\sum_{i=1}^{n}\xi_{i}-b\\ s.t.\;\left(wx_{i}\right)>b-\xi_{i},{\;\xi }_{i}\geq 0,\;i=1,2,\cdots ,n \end{array}$$

where *c* is the penalty parameter with the value range (0, 1), *n* is the number of samples, *ξ* is the relaxation variable with a value greater than 0, and *b* is the intercept of the hyperplane. Combined with the Lagrange book penalty, the discriminant function of the classification model is finally obtained as follows:

(6) $$f(x)=\mathrm{sgn}\left(\overset{n}{\underset{i=1}{\sum }}a_{i}K\left(x,x_{i}\right)-b\right)$$

where *K*(*x*, *x_i_*) is the kernel function, *Q* is the control frequency, the formula is as follows:

(7) $$K\left(x,x_{i}\right)={\left(x\cdot x_{i}+1\right)}^{Q}$$

Considering that the signal parameters have highly coupled features, in order to extract key characteristic values and save computing power, the principal component analysis (PCA) feature extraction method is used to extract the characteristic values [33]. Since the dimensions of eigenvalue parameters are different, direct input to the model will affect the output result of the model, so the maximum or minimum value is used to normalize them.

To compare and verify the view that this ski pole can improve the accuracy of ski pattern recognition, during the test, when three athletes use the ski poles provided by us to ski, we will install the IMU on the arms and the back and neck parts at the same time, so as to compare the differences based on three different wearing methods. The skiers followed five different ski techniques, including three traditional cross-country techniques, Diagonal Stride (DS), Double Poling (DP), Kick Double Poling (KDP), and two freestyle cross-country techniques, Two-stroke Glide (G2) and Five-stroke Glide (G5), each with a 3–4 min slide to ensure sufficient data. Of these, DS involves the skier gliding forward by alternately moving the opposite arm and leg, DP involves the skier pushing and gliding forward with two poles simultaneously, KDP combines leg kicks with simultaneous pushing of both poles to gain forward momentum, G2 requires the skier to perform two pole pushes with each leg kick, G5 requires the skier to perform five pole pushes with each leg kick to maximize speed and efficiency. The decomposition diagram of the five actions is shown in Figure S1.

## 3. Result

### 3.1. Difference Comparison

The *t*-test was utilized to analyze whether there were significant differences in the data among the three individuals. Before conducting the sample *t*-test, the data underwent a normality test, the results of which indicated that, at the 0.01 significance level, the data significantly originated from a normal distribution overall. As shown in Figure 3d–f, comparisons were made between the peak pole thrust pressure, insertion angle, pole thrusting time, and skiing time of the three skiers, with a significance level set at *p* = 0.01 to determine the presence of extremely significant differences, where ‘*’ means p≤0.05 and ‘**’ means p≤0.01. Comparing the three test participants, significant differences were observed in their pole acceleration, pole angle, pole time, and skiing time. As shown in Figure 3b, Participant 3 reached top speed, reaching a maximum speed of 12.4 km/h during the course of the slide within 1000 m. Specifically, his skiing technique exhibited the following features:

(1) Larger pole angles: allowing for more effective utilization of the ski poles to achieve higher speeds.
(2) Shorter pole time: indicating faster pole insertion speed and shorter pole duration.
(3) Stronger pole force: suggesting he might exert greater force to propel himself forward.
(4) Shorter cycle length: indicating he might engage in pole planting more frequently to maintain speed.

In conclusion, the analysis of skier data reveals technical differences among different skiers. This information can be used to enhance skiing techniques and provide valuable insights into improving speed and efficiency. These findings hold practical significance for both skiers and coaches.

Analyzing the ski pole data during steady skiing, a correlation analysis was conducted on pressure, angle, acceleration, and speed to verify the previous conclusions. As shown in Figure 3g, the following conclusions can be drawn from the correlation results:

(1) The correlation coefficient between pressure and speed is 0.71, demonstrating a significant moderate correlation. This suggests that there is a certain correlation between pressure and speed during skiing, possibly indicating that ski pole pressure varies correspondingly when skiers accelerate or decelerate.
(2) The correlation coefficient between ski pole angle and pressure is 0.76, also exhibiting a significant moderate correlation. This indicates a close association between ski pole angle and applied pressure, potentially influenced by the position of the ski poles. Different ski pole angles may affect the magnitude of pressure applied by skiers.
(3) The correlation coefficient between acceleration and axial load force is 0.55, showing a moderate correlation. This phenomenon arises from the drastic positive and negative changes in acceleration during ski pole–ground contact, as well as the large acceleration during recovery actions, resulting in a moderate correlation without clear positive or negative characteristics.
(4) The correlation coefficient between acceleration and angle is 0.324, demonstrating a moderate correlation. Due to the abrupt nature of acceleration, its relationship with angle is not as pronounced.

In summary, the above conclusions reveal the correlations between different factors during skiing, thereby validating the previous findings that factors such as pressure, pole angle, and pole frequency directly influence speed. These results further emphasize the complex relationships among various factors in skiing and their significance for overall skiing experience and performance. A deeper understanding of these correlations can assist skiers in adjusting their technical movements and strategies, thereby enhancing skiing efficiency and performance.

### 3.2. Action Recognition

Data from ski poles and roller skis are analyzed in Figure 4b. There are some slight differences between the left and right ski poles. The right-hand pole appears stronger, and the pole frequency on both sides appears roughly the same. Additionally, the data on roller skis did not vary significantly, which proves that the skiers were sliding steadily. Observing the z-axis gyroscope data in Figure 4b, it can be found that there is an obvious sinusoidal variation rule of this waveform during the ski pole cycle action. Based on this change in the z-axis gyroscope data, a complete action cycle is extracted for analysis. To gain a better understanding of skiing movements, we have divided them into distinct stages: (1) the contact instant, (2) the propulsion stage, and (3) the ski recovery stage, as shown in Figure 4c. These stages are represented in Figure 4d using video frames with corresponding colors. The orange frame corresponds to the moment when the ski pole makes contact with the ground, marking the start of the action. The skier pushes the poles backwards to accelerate from the orange to the green frame. Finally, the transition from the orange to the purple frames indicates the end of the pole action, with the skier gradually returning to the starting position. This cycle allows the skier to either accelerate or maintain their speed.

**Figure 5:**
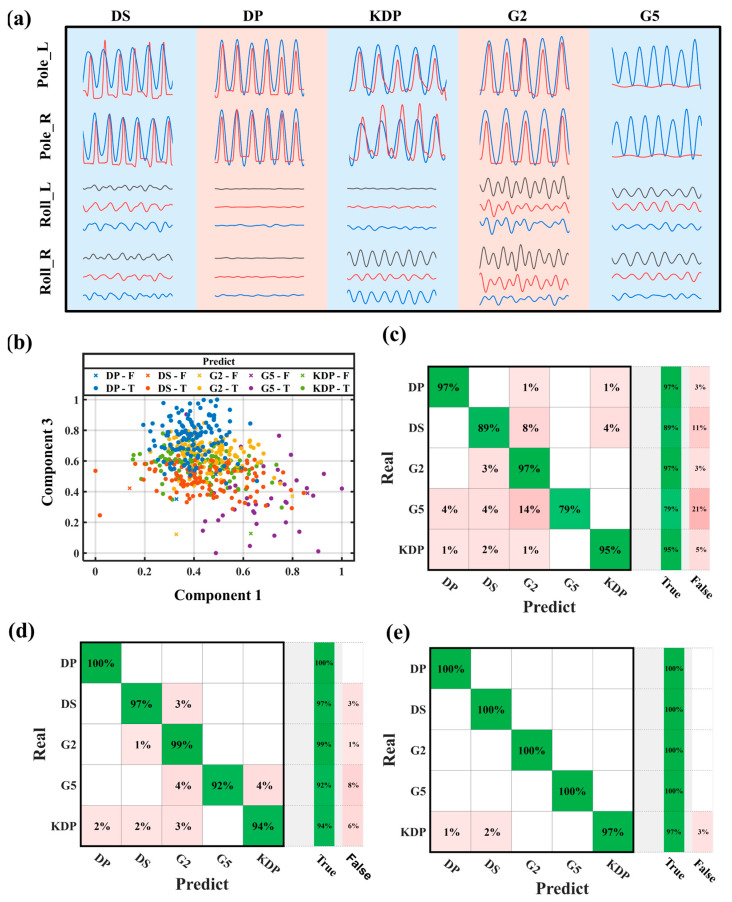
Classification and identification of five cross-country skiing movements. (**a**) Time domain waveform of five cross-country skiing movements. (**b**) Scatter diagram and (**c**–**e**) confusion mapping of pattern recognition results was performed using SVM.

In Figure 5a, data of multiple cycles (more than five cycles) of five kinds of actions are drawn, and five different technologies are clearly defined. It can be observed that the time-domain waveform of each technical action has certain differences. The experimental data included a total of 575 technical cycles, of which G2 accounted for 131 cycles, G5 accounted for 50 cycles, DP accounted for 137 cycles, DS accounted for 141 cycles, and KDP accounted for 116 cycles. For minority sample data, the Synthetic Minority Over-sampling Technique (SMOTE) algorithm generates synthetic samples to balance the class distribution. To prevent overfitting, the training data set is divided into ten samples using cross-validation, and the accuracy of each sample is estimated. Nine samples are randomly selected as the training set, and the remaining one is used as the test set. After the completion of one round, the training set and the test set are randomly re-combined, and the confusion matrix can be obtained after several rounds.

To train the classifier, we used the LibSVM software package in MATLAB (R2024a). Figure 5c–e display the confusion matrix for the triple SVM training results. The model is 93.7%, 97.7%, and 99.5% accurate, respectively. According to the trial findings, the smart ski pole combined with an inertial sensor unit and an axial load unit improves the skiing technology’s ability to recognize patterns.

## 4. Discussion

The smart ski pole used in this work incorporates inertial and pole force measurements, and its integrated design and wireless transmission mode make it more appropriate for field measurements and recording in an outside setting. The ski poles have a sampling rate of 60 Hz, and the transmission rate is stable and effective at 57,600 baud when the antenna is installed at the receiver. At this high transmission rate, data loss minimally impacts the experimental results. Compared to the rigid or flexible sensors attached to each joint of the skier by Fasel et al. [28,29] and Yang et al. [27], the intelligent ski pole has almost no impact on the skier during the monitoring process. Its weight can be adjusted to match the feel of a regular ski pole.

The ski poles can be used to measure several key pole features during cross-country skiing. In this experiment, data on the characteristics of the snow poles during bipolars of three skiers were extracted, and it can be found from the tests and analyses that the pole force and speed (*r* = 0.71) and pole support angle (*r* = 0.76) were significantly correlated. We demonstrated the part discussed by Russo et al. [26], showing that the force applied by ski poles has a strong correlation with skiing speed and is essential for information acquisition. It is worth noting that when using the ski poles as an important part of the action recognition system, the recognition accuracy of the five cross-country skiing technical actions in this experiment reaches 99.5%. This system is convenient to wear and surpasses the 98.2% accuracy achieved by Yang et al. [27] for recognizing four techniques. And compared to similar recognition systems, the accuracy is significantly improved.

However, the study has certain limitations that need to be addressed in future research. Firstly, although the three participants are representative, a larger sample size is required to validate the results. Secondly, due to site constraints, we only simulated flat sections rather than the entire cross-country skiing process. Future studies should develop more complex analytical models that encompass different skiing stages. Nevertheless, the portability and monitoring capabilities of the ski poles and their measurement systems ensure their viability for ski training applications, and the technology of integrating multi-sensor and wireless transmission modules promotes the future development of portable performance measurement tools. With the advancement of technology and the demand of the market, the future may see the emergence of more portable, practical, and affordable solutions, so that these devices can be more widely used in all levels of sports training.

## Figures and Tables

**Table 1:** Table of the demographic data.

Subjects	Height (cm)	Weight (kg)	Age	Maximum Speed (km/h)
Participant (1)	185	80	25	9.7
Participant (2)	182	82	25	10.5
Participant (3)	180	79	25	12.4

**Table 2:** Table of characteristic values.

Eigenvalue			
Maximum	Standard deviation	Root mean square factor	Barycentric frequency
Minimum	Kurtosis	Peak factor	Mean square frequency
Mean	Skewness	Pulse factor	Frequency variance
Peak	Root mean square	Margin factor	

## Data Availability

The original contributions presented in the study are included in the article/Supplementary Materials, further inquiries can be directed to the corresponding authors.

## Supplementary Materials

The following supporting information can be downloaded at: https://www.mdpi.com/article/10.3390/s24165291/s1, Figure S1: (a–e) The decomposition diagram of DS, DP, KDS, G2, G5, respectively; Video S1: Function demonstration of intelligent ski pole’s grip; Video S2: Ski training demonstration based on the intelligent ski pole.
