# Supplementary material for: A Smart Ski Pole for Skiing Pattern Recognition and Quantification Application
Source: Sensors (Basel). 2024 Aug 15;24(16):5291. doi: 10.3390/s24165291 (PMC11360248; doi:10.3390/s24165291)
Supplement: Supplementary file 1 [file sensors-24-05291-s001.zip › Supporting information.pdf]

sensors

MDPI

Supporting Information for

A Smart Ski Pole for Skiing Pattern Recognition and Quantification Application

Yangyanhao Guo [1], Renjie Ju [1], Kunru Li [1], Zhiqiang Lan [2,*], Lixin Niu [1], Xiaojuan Hou [1], Shuo Qian [3], Wei Chen [1], Xinyu Liu [1], Gang Li [4], Jian He [1,*] and Xiujian Chou [1]

1 Science and Technology on Electronic Test and Measurement Laboratory, School of Instrument and Electronics, North University of China, Taiyuan 030051, China; s202206044@nuc.edu.cn (Y.G.); sz202206019@nuc.edu.cn (R.J.); s202306056@st.nuc.edu.cn (K.L.); sz202106125@nuc.edu.cn (L.N.); houxiaojuan@nuc.edu.cn (X.H.); sz202106192@nuc.edu.cn (W.C.); sz202106136@nuc.edu.cn (X.L.); chouxiujian@nuc.edu.cn (X.C.)
2 School of Future Science and Engineering, Soochow University, Suzhou 215299, China
3 School of Software, North University of China, Taiyuan 030051, China; qianshuo@nuc.edu.cn
4 School of Physical Education , Tianjin University of Sport, Tianjin 301600, China; lig1117@126.com
* Correspondence: zqlan@suda.edu.cn (Z.L.); drhejian@nuc.edu.cn (J.H.)

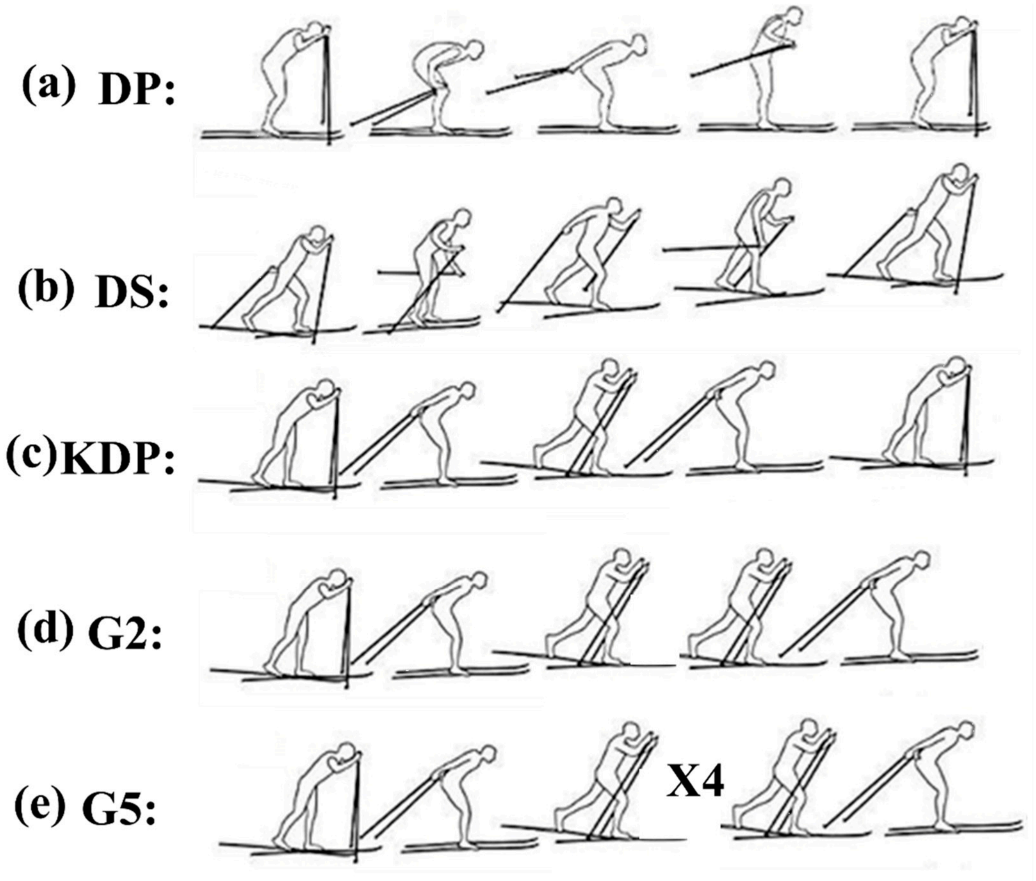

Figure S1. (**a**–**e**) The decomposition diagram of DS, DP, KDS, G2, G5, respectively.

Video S1. Function demonstration of intelligent ski pole's grip.

Video S2. Ski training demonstration based on the intelligent ski pole.
